# High sacral slope, lumbar lordosis, and sacral slope-to-pelvic incidence ratio are associated with new bone formation in ankylosing spondylitis

**DOI:** 10.55730/1300-0144.5915

**Published:** 2024-10-25

**Authors:** Aysun AKSOY, Cemal Aydın GÜNDOĞMUŞ, Mehmet Deniz KESİMER, Kemal NAS, İkram Eda DUMAN, Gazanfer EKİNCİ, Murat BEZER, Pamir ATAGÜNDÜZ

**Affiliations:** 1 Department of Rheumatology, Faculty of Medicine, Marmara University, İstanbul, Turkiye; 2 Department of Radiology, Faculty of Medicine, Marmara University, İstanbul, Turkiye; 3 Department of Orthopedic and Traumatology, Faculty of Medicine, Marmara University, İstanbul, Turkiye; 4 Department of Physical Therapy and Rehabilitation, Faculty of Medicine, Sakarya University, Sakarya, Turkiye

**Keywords:** Ankylosing spondylitis, lumbar lordosis, pelvic incidence, sacral slope, syndesmophyte

## Abstract

**Background/aim:**

Syndesmophyte formation appears to be site-specific in ankylosing spondylitis (AS) and new bone formation seems to occur in regions of microtrauma that are prone to tensile forces. Pelvic and spinal parameters are unique for each individual. Pelvic tilt and sacral slope are important anatomical features that compensate in harmony in keeping the sagittal balance. After puberty, the sacral slope shapes the lumbar lordosis, whereas the pelvic incidence has an individual constant value. This study aimed to analyze the properties of pelvic parameters in AS patients with and without syndesmophyte formation in the spine after 15 years of disease duration.

**Materials and methods:**

Whole-spine radiographs and clinical data of 104 AS patients were analyzed according to radiographic damage in the spine. AS patients were grouped as those with and without syndesmophytes. Patients with complete bridging in at least one vertebral unit were excluded. Sacral slope, pelvic tilt, pelvic incidence, and lumbar lordosis were measured.

**Results:**

The mean disease duration was 14.5 years and 60% of the AS patients were male. The groups were similar in terms of age, sex distribution, and disease duration. Although numerically higher in patients with syndesmophytes, the mean pelvic incidence of AS patients was not significantly different between groups (55.2 ± 13.6 vs. 57.2 ± 15.4). The sacral slope was higher in patients with lumbar syndesmophytes (p < 0.005).

**Conclusion:**

The sacral slope was significantly higher in patients with syndesmophytes, which in turn resulted in increased lumbar lordosis. Our results imply that the individual shape of the spine affects the distribution of weight and tensile forces in AS, and some patients are possibly more prone to new bone formation due to altered repetitive microtrauma in the general genetic background of AS. Prospective studies addressing this cross-sectional observation may contribute to the development of new treatment strategies addressing mechanical load and may aid in decreasing the management costs of AS with the present biological therapies targeting new bone formation.

## 1. Introduction

Chronic inflammation of the entheses, mainly of the sacroiliac joints and the spine, characterizes ankylosing spondylitis (AS). Patients developing syndesmophytes and heavy functional loss in the early years of the disease are more likely to experience disease progression [1]. Despite intensive research, the mechanisms of the scattered anatomical distribution of inflammation and new bone formation in AS throughout the vertebral column is not clear. However, it is generally accepted that new bone formation occurs in regions that are more prone to tensile forces [2]. Jacques et al. reported the significance of repetitive microtrauma in the development of inflammation at sites of enthesis. The scattered distribution of syndesmophytes when they first appear in the vertebral column may imply that forces affecting the vertebral units influence the location of inflammation and possibly the development of syndesmophytes [3].

Pelvic and spinal parameters are used to define the shape of the spine, which is unique for each individual. Pelvic incidence assumes a constant and individual value after puberty. In contrast, pelvic tilt and sacral slope are dynamic parameters that compensate in harmony to keep the sagittal balance in the setting of that individual’s constant pelvic incidence. Therefore, after puberty, the sacral slope plays a critical role in determining lumbar lordosis and the distribution of the forces affecting the single vertebral units [4–6].

Previously, based on the sacral slope, four different shapes of the vertebral column were described by Roussouly and Pinheiro-Franco, and the relationship of lumbar lordosis and thoracic kyphosis in an individual was shown to affect the horizontal and vertical forces on the vertebrae [7]. Similarly, the importance of the variance of the spinopelvic parameters on degenerative diseases such as osteoarthritis, disc herniation, and spondylolisthesis or spondylolysis were previously described in the literature [8–10]. Whether unfavorable values of the pelvic parameters may have an impact on new bone formation in the spine is not known.

In this study, we aimed to characterize the pelvic parameters of AS patients categorized within two distinct groups with different radiographic progression but similar disease duration.

## 2. Materials and methods

Demographic and clinical data from 242 consecutive AS patients followed between April 2017 and December 2018 in our outpatient clinics were analyzed retrospectively. All patients met the modified New York criteria (mNY) [11]. Enrolled patients were between the ages of 18 and 50 years. Patients with neurological or psychiatric diseases, inborn or acquired orthopedic diseases of the spine (scoliosis, spinal surgery, internal fixation), and hip or knee prostheses were excluded. Patients with ≥3 missing values for the modified Stoke Ankylosing Spondylitis Spinal Score (mSASSS) were also excluded from the analysis.

**Figure 1:**
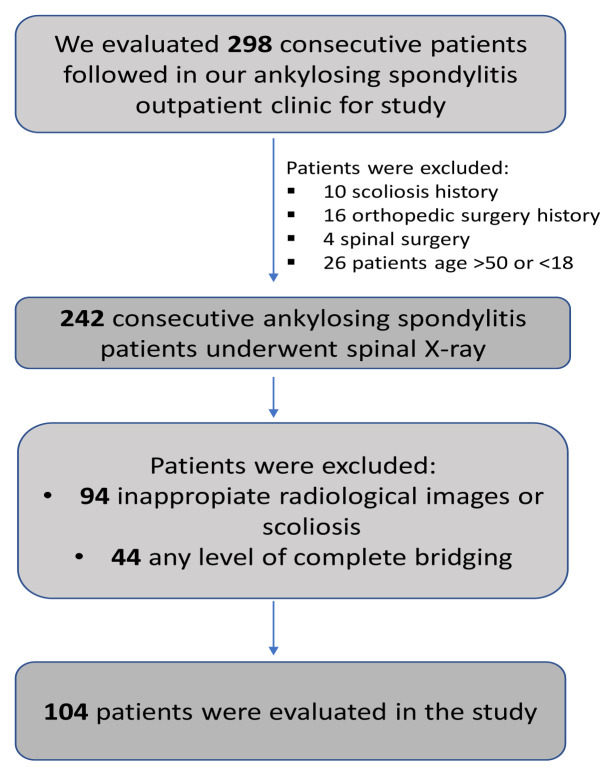
Lateral whole-spine radiograph of a patient with ankylosing spondylitis.

**Figure 2:**
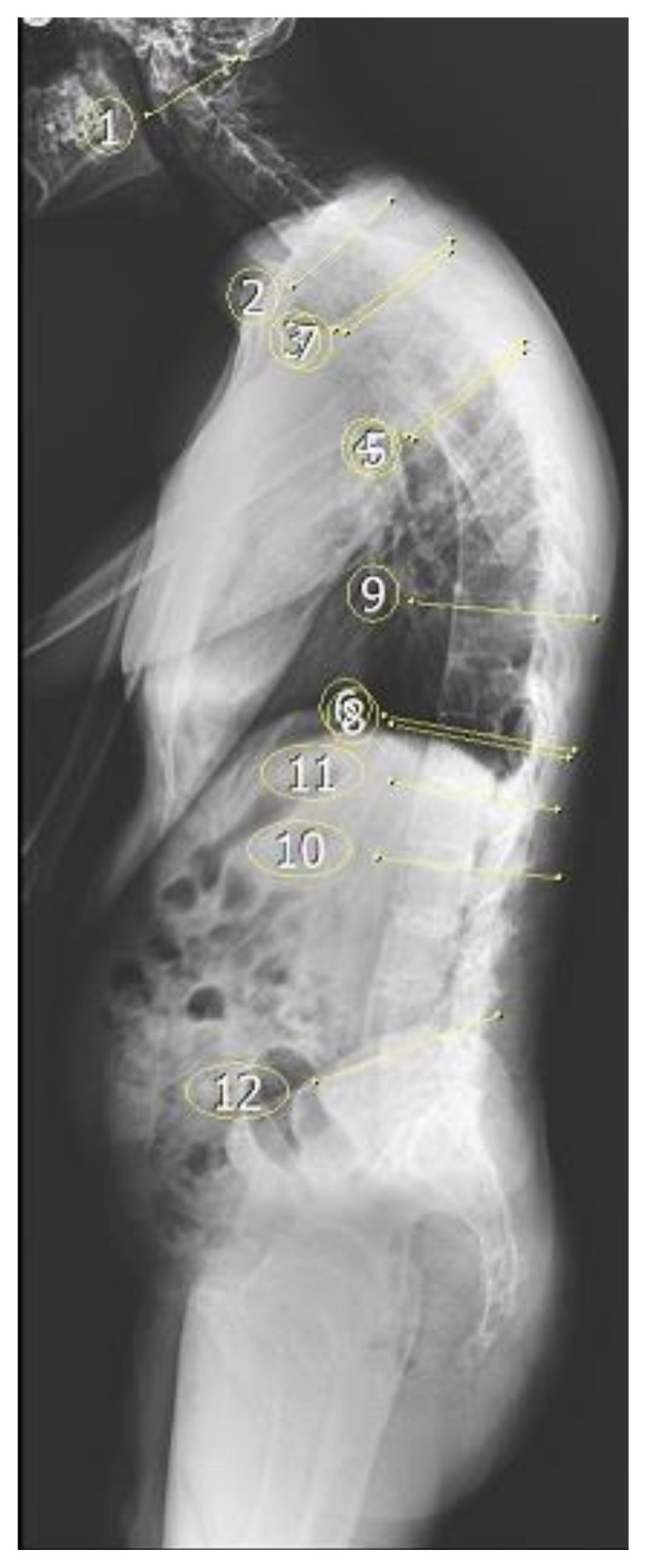
Flow chart of the study process.

Participants underwent routine anteroposterior and lateral whole-spine radiographs to reveal a possible mechanical influence of anatomical origin due to spinal pain (Figure 1). Radiographs were taken by the same technician with a standard technique in the standing position using the same machine. The eligibility of lateral radiographs for standard measurements of spinopelvic parameters and correct positioning of both hip joints and the C7 vertebra on radiographs was assessed by an orthopedic surgeon and a radiologist. The radiographs of 94 patients were excluded for inappropriate images or scoliosis (Cobb angle of >10°) and this assessment resulted in 148 eligible patient files for the study. We excluded 44 patients with differing degrees of complete bridging or bamboo spine to eliminate the effect of the immobile vertebral segments on spinopelvic parameters. After that exclusion, we continued to include data from consecutive AS patients until we had obtained two groups of patients with equal distributions of age, sex, and disease duration. The flow chart provided in Figure 2 displays the study process. A total of 104 patients were included in the final analysis.

Disease duration was calculated from the beginning of the patient-reported inflammatory back pain to the study time. Patients’ occupations were classified as follows: unemployed, blue collar (jobs that require primarily physical activity, such as laborers, waitresses, etc.), or white collar (jobs that require primarily mental effort, such as lawyers, civil servants, etc.).

### 2.1. mSASSS scoring and patient groups

Lateral whole-spine radiographs were assessed by two experts who were blinded to the patients’ clinical data. mSASSS scoring was done as previously described. Briefly, each anterior corner of the cervical spine (from the lower corner of the C2 vertebra to the upper corner of the T1 vertebra) and the lumbar spine (from the lower corner of the T12 vertebra to the upper corner of S1 vertebra) on lateral radiographs was scored between 0 and 3 (0 = no abnormality; 1 = erosion, sclerosis, or squaring; 2 = syndesmophyte; 3 = bridging syndesmophyte), with total scores ranging from 0 to 72 [12].

The median mSASSS score of our study group was 6 (range: 0–20). Interobserver error for mSASSS scoring was calculated.

The first group of patients had no syndesmophytes at all and constituted the mild progression group, designated as patients with no syndesmophytes (“no syndesmophytes group”). The second group of patients had one or more syndesmophytes but no complete bridging of the vertebral units, designated as patients with syndesmophytes (“syndesmophytes group”).

Radiographic measurements were done for the following parameters: sacral slope, pelvic tilt, pelvic incidence, and lumbar lordosis. **The sacral slope** was defined as the angle between the sacral endplate and the horizontal. The sacral plateau forms the base of the spine and so the degree of the slope determines the position of the lumbar spine. **Pelvic tilt** was defined as the angle between the line joining the middle of the sacral endplate and the hip axis and the vertical line. **Pelvic incidence** was defined as the angle formed by the intersection of the line drawn from the center of the femoral heads to the middle of the sacral plate and the line running perpendicular to the middle of the sacral plate. The pelvic incidence is the angle that describes the relationship between the sacral plate and the femoral heads. It is a constant morphological parameter for an individual after skeletal maturity is reached in adulthood **(**F**igure 3)**. Finally, lumbar lordosis was measured between the upper endplate of the L2 vertebra and the lower endplate of the L5 vertebra using Cobb’s method.

All measurements were performed twice independently by an orthopedic surgeon and a radiologist with an interval of 2 weeks between measurements to decrease interobserver error. The Pearson correlation coefficient was 0.845 (range: 0.762–0.900).

The study was approved by the Clinical Research Ethics Committee of our institution (09.2017.521).

### 2.2. Statistical analysis

Categorical variables were presented as frequencies and percentages. Continuous variables were tested for the assumption of normality with histograms, normal quantile plots, Kolmogorov–Smirnov tests, and skewness and kurtosis tests. The continuous variables without normal distribution (CL, T10–L2 kyphosis, and mSASSS) were presented with median, 25th–75th quartile, and minimum and maximum values. The continuous variables were compared between the groups using Mann–Whitney U and Kruskal–Wallis tests. Correlations between continuous variables were evaluated with the Spearman test and both rho and p values were reported. All tests were two-sided and values of p < 0.05 were considered significant. Analyses were performed using IBM SPSS Statistics 21 (IBM Corp., Armonk, NY, USA).

## 3. Results

Of the 104 AS patients, 42 (40.4%) were female and 62 (59.6%) were male. The mean age was 39.9 ± 6.2 years and the mean disease duration was 14.5 ± 7 years. The mean disease duration, sex distribution, and mean age were similar in the two radiographic groups (p > 0.05). Table 1 summarizes the demographic characteristics of the study population.

The mean age and disease duration, history of smoking and uveitis, percentage of HLA-B27 positivity, and mean mSASSS did not differ between the sexes (p > 0.05). At enrollment, male patients were treated more frequently with tumor necrosis factor inhibitors (72.7% vs. 42.9%, p = 0.008).

Table 2 summarizes the pelvic parameters of the analyzed patients. The mean sacral slope was higher in patients with syndesmophytes compared to the no-syndesmophytes group. Similarly, the mean sacral slope was significantly higher in patients with syndesmophytes compared to the no-syndesmophytes group for male AS patients (43.5 ± 11.8 vs. 37.6 ± 9.9, p = 0.043). However, the mean sacral slope did not differ between the groups for female patients (37.4 ± 11.6 vs. 42.3 ± 8.3, p = 0.079).

Patients with or without cervical syndesmophytes (independently of having a syndesmophyte in the lumbar area) had similar pelvic incidence, sacral slope, pelvic tilt, and lumbar lordosis (p > 0.05), while patients with at least one lumbar syndesmophyte had increased sacral slope compared to patients without syndesmophytes in the lumbar region (45.6 ± 11.2 vs. 38.1 ± 10, p = 0.005).

We also observed a positive correlation between mean sacral slope and number of syndesmophytes in the lumbar region (r = 0.276, p = 0.005).

## 4. Discussion

The primary endpoint of this study was the characterization of pelvic parameters of AS patients in relation to the radiographic burden of the spine. The mean pelvic incidence in the AS patients of our study group was 55°, a value higher than those reported for healthy individuals in previous studies. A large study from Türkiye addressing normal spinopelvic parameters reported a mean pelvic incidence value of 47.4°, which is substantially lower than the pelvic incidence in our study population, while the pelvic incidence values of other previous studies are also higher among AS patients [13–15].

AS is an inflammatory disease characterized by new bone formation and a recent proof-of-concept study proposed that repeated microtrauma due to altered weight-bearing may lead to enthesis and new bone formation in AS [3].

Another important observation of our study is that sacral slope and lumbar lordosis deviated significantly in AS patients who developed syndesmophytes after the same disease duration (15 years) as those without radiographic damage. Patients with no syndesmophytes had a mean sacral slope value of 37°, significantly lower compared to those with syndesmophytes, and this finding coincides with the previously suggested classification of the “harmonious regular back,” which is largely protected from degenerative spine diseases [7].

Our novel approach of defining the shape of the vertebral column in AS and incorporating the concept of repeated trauma due to a disadvantageous weight distribution throughout the spine may be advantageous in understanding the scattered nature of syndesmophyte distribution in the spine. Despite the cross-sectional nature of this study, our results imply the role of a high pelvic incidence value in the development of radiographic damage, and especially in the lumbar region, after 15 years of disease duration.

Pelvic incidence, which is unique for each individual and has a presumably constant value after puberty, reflects the orientation of the sacrum inside the pelvis [5]. The reciprocal compensation of the sacral slope and pelvic tilt is limited by pelvic incidence. The importance of our observation lies in the fact that the sacral slope is the main determinant of lumbar lordosis and, similar to the predisposition to degenerative spine diseases, AS patients with similar general genetic backgrounds but distinct anatomical features of the spine are possibly more prone to new bone formation in the lumbar region of the spine [7].

A high sacral slope and a tendency towards higher lumbar lordosis in the presence of high pelvic incidence in the AS patients with syndesmophytes implies that inborn anatomical variances may affect the radiographic burden in AS. We observed that the mSASSS for the lumbar region was positively correlated with sacral slope and pelvic incidence in our study population. The strongest correlation was observed for sacral slope, as seen in Table 2. Sacral slope itself and the ratio of sacral slope to pelvic incidence were significantly lower in the no-syndesmophytes patient group and lumbar lordosis was affected. Therefore, we suggest that the shape of the vertebral column may affect the weight distribution on vertebral units by altering the dynamics of the tensile forces acting on vertebral units, causing the scattered pattern of syndesmophytes throughout the spine. It is already known that different types of degenerative diseases are associated with different types of spines with distinct mechanical harmonics [7].

The previous prospective observations of Ramiro et al. [16] revealed that one-quarter of AS patients did not show any radiographic progression after 12 years of follow-up and approximately 40% of all patients did not develop a new syndesmophyte in that time period. Accordingly, they concluded that new bone formation is highly variable and to a large extent unpredictable in the course of the disease and individual differences in new bone formation are unique to each AS patient [16]. However, in these well-designed studies, spine morphology in association with radiographic burden was not controlled. Our patient group with syndesmophytes and without syndesmophytes all met the mNY criteria and had similar disease characteristics and durations as those described in the above-mentioned study. Assessing the shape of the spine initially and repeated measurements of the pelvic parameters could also explain the different rates of radiographic progression in individual AS patients at different stages of their lives or over the course of the disease. As suggested previously by Legaye [17] and Bao et al. [18], degeneration of the weight-bearing spinal elements with increasing age may affect the rate of new bone formation in individual AS patients and may imply acceleration in radiographic progression after 10 to 15 years of mildly progressing disease, as seen in the well-followed AS patient group reported by Ramiro et al. [16].

One limitation of our study is its cross-sectional nature. We did not have an initial set of values for spinopelvic parameters of the analyzed patients. However, while there is a consensus that pelvic incidence remains constant throughout life after attaining skeletal maturity [5], some previous reports argue that a change in pelvic incidence may be observed under certain conditions in the elderly. Legaye [17] proposed that sacroiliac joint degeneration as a consequence of aging and sagittal malalignment can directly lead to an increased pelvic incidence. Bao et al. [18] later confirmed the effect of age by reporting an increase in pelvic incidence after the age of 60 years.

On the basis of our results, one may suggest that, together with medical treatment, exercise to increase muscle strength and improve the shape of the spinal curvature is critical in the management in AS. When patients with AS were compared with healthy populations, a reduction in the strength of the quadriceps was found in several studies with different methods such as isokinetic tests and quantitative surface electroneuromyography. It is known that there is a tendency for knee flexion and ankle plantar flexion posture in patients with AS, but plantar flexor muscle strength reduction was found in a study based on isokinetic test assessments [19].

A multimodal supervised approach through exercises, physiotherapy, patient education, and other nonpharmacological interventions may help in improving disease activity, functional capacity, and pain in patients with AS [20]. In light of our results, studies investigating the effects of exercise in the long term, especially in mobilizing and shaping the normal curvature of the spine, with regard to radiographic progression may be of interest.

Our study had some limitations. As noted above, the study was cross-sectional and so we did not have the spinopelvic parameters of the patients at the beginning of the disease or prospective follow-up data of our patient groups. Furthermore, we had results only from standing radiographs of the spine and so we were not able to evaluate the interaction of dynamic features of the spine with syndesmophytes. AS was chosen in our study as a prototype disease with spinal involvement to assess spinopelvic parameters, although we may speculate that other diseases causing inflammation in the spine resulting in syndesmophytes would show similar spinopelvic parameters changes. Further research is needed to support this hypothesis.

In conclusion, the high pelvic incidence of AS patients seems to affect the sacral slope and lumbar lordosis, implying that the individual shape of the spine may predestine an AS patient’s radiographic burden. In light of recent reports emphasizing the role of mechanical burden on new bone formation in AS, the inborn shape and weight distribution seems to be gaining importance in the management of AS patients. Increased sacral slope and lumbar lordosis and increased sacral slope/pelvic incidence ratio seem to be critical for an inappropriate weigh distribution in the spine and may be responsible for repetitive strain. Along with expensive medical therapy, the role of a multimodal supervised approach involving exercises and physiotherapy with the aim of posture correction in the spine should be addressed in studies involving radiographic progression in AS. We believe that it may be advisable for future prospective studies addressing the etiopathogenetic of radiographic progression that pelvic parameters be included as independent prognostic factors.

## Figures and Tables

**Figure 3:**
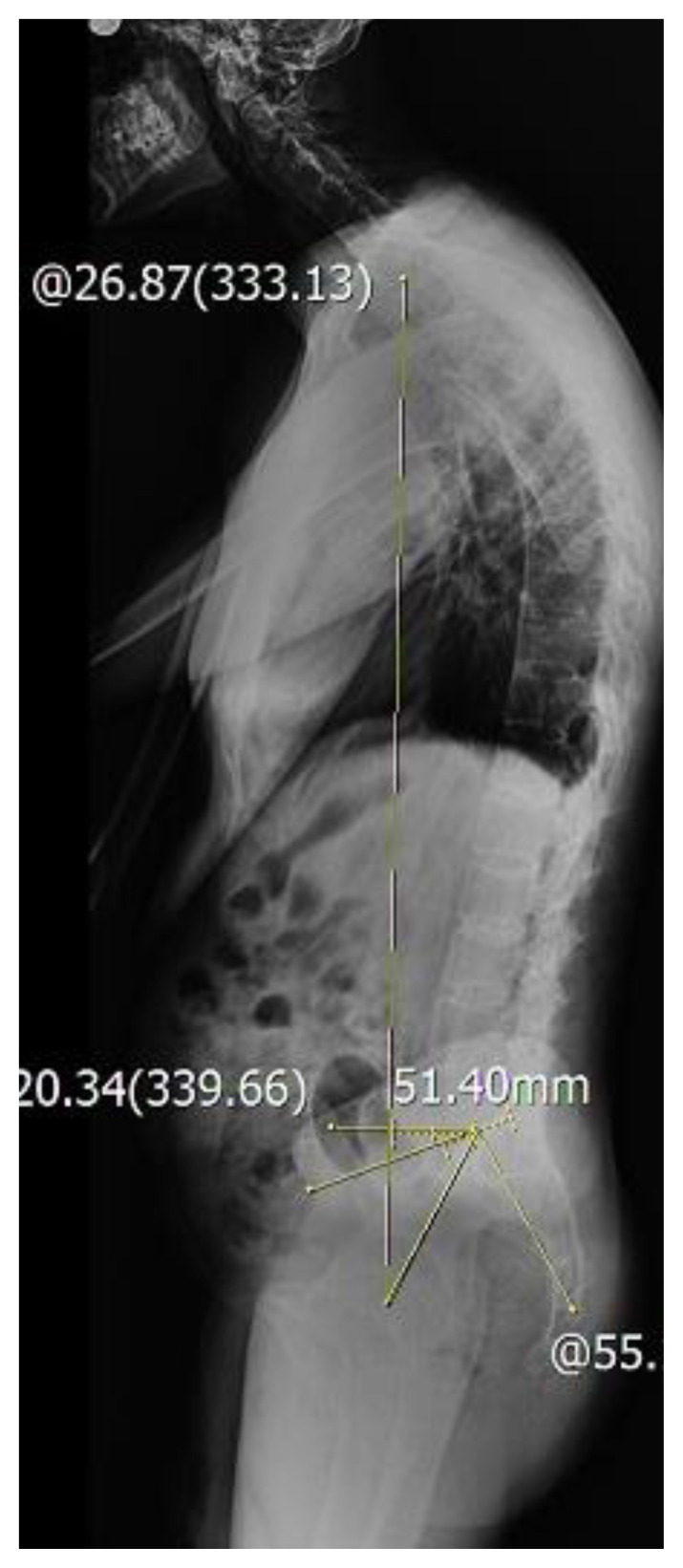
Measurement of pelvic incidence, sacral slope, and pelvic tilt.

**Table 1:** Demographic and clinical characteristics of the study population.

		Whole group	No-syndesmophytes	Syndesmophytes	p
Sex (male), %		62, 59.6%	36, 61%	26, 57.8 %	.841
Age		39.9 ± 6.2	39 ± 6.3	41.1 ± 6	.087
Body mass index		27.2 ± 4	27.4 ± 4.4	26.9 ± 3.6	.524
Occupation	No occupation	32 (31.4%)	18 (31.6%)	14 (31.1%)	.999*
	Blue collar	53 (52%)	31 (54.4%)	22 (48.9%)	.576**
	White collar	17 (16.7%)	8 (14%)	9 (20%)	.564***
HLA B27 positivity, %		59, 62.1%	31, 58.5%	28, 66.7%	.524
Uveitis history, %		17, 16.7%	9, 15.5%	8, 18.2%	.792
Smoking, %		49, 47.6%	39, 39.7%	26, 57.8%	.077
TNFi usage, %		54, 60.7%	28, 53.8%	26, 70.3%	.130

* no occupation versus blue collar,

** blue collar versus white collar,

*** no occupation versus white collar

HLA: human leucocyte antigen, TNFi: tumor necrosis factor inhibitors

Number, percentage. Mean ± standard deviation

**Table 2:** Spinopelvic parameters of the study population.

Whole study population	Whole group (n:104)	No-syndesmophytes (n: 59)	Syndesmophytes (n: 45)	p
Pelvic incidence	55.2 ± 13.6	53.6 ± 12	57.2 ± 15.4	.205
Sacral slope	39.9 ± 10.7	37.5 ± 10.5	43 ± 10.4	**.009**
Pelvic tilt	14.3 (9.4–20)	15.1 (10.2–20)	12.9 (7.3–20.1)	.220
Lumbar lordosis	62 ± 12.3	60.2 ± 13.2	64.5 ± 10.5	**.067**
SS/PI	0.73 ± 0.13	0.70 ± 0.14	0.76 ± 0.11	**.018**
SS/LL	0.64 ± 0.12	0.63 ± 0.13	0.67 ± 0.1	.105
PI/LL	0.91 ± 0.25	0.92 ± 0.3	0.89 ± 0.17	.430

LL: lumbar lordosis, PI: pelvic incidence, SS: sacral slope

mean± standard deviation
